# Non‐invasive adhesive patch microRNA assay recapitulates tissue biomarkers for melanoma

**DOI:** 10.1002/ctm2.70627

**Published:** 2026-02-15

**Authors:** Carly A. Becker, David W. N. Mwelwa, Amanda Z. Jiang, Annabelle Huntsman, Kenneth M. Boucher, Chris J. Stubben, Eric A. Smith, Dekker C. Deacon, Douglas Grossman, Robert L. Judson‐Torres

**Affiliations:** ^1^ Huntsman Cancer Institute University of Utah Salt Lake City Utah USA; ^2^ Department of Dermatology University of Utah Salt Lake City Utah USA; ^3^ Department of Oncological Sciences University of Utah Salt Lake City Utah USA; ^4^ Spencer Fox Eccles School of Medicine University of Utah Salt Lake City USA; ^5^ Department of Internal Medicine Huntsman Cancer Institute University of Utah Salt Lake City Utah USA; ^6^ Cancer Bioinformatics Shared Resource, Huntsman Cancer Institute University of Utah Salt Lake City Utah USA; ^7^ Department of Pathology University of Utah Salt Lake City Utah USA

1

Dear Editor:

We show that microRNAs (miRNAs) are substantially more stable than long RNAs in tape‐stripped skin samples and can be robustly quantified by digital polymerase chain reaction (dPCR). Using this approach, a miRNA ratio previously validated in tissue specimens retains its ability to distinguish melanocytic nevi (MN) from invasive melanoma (IM) when measured non‐invasively.

Many clinically suspicious pigmented skin lesions are biopsied to exclude melanoma, yet the vast majority prove to be benign (e.g. MN), resulting in substantial over‐biopsy and patient burden^1^. This reflects an unmet need for objective, molecular pre‐biopsy triage tools to help prioritise lesions for histopathologic evaluation. Existing non‐invasive approaches, including image‐based and RNA‐based tape stripping (TS) assays, have shown promise but are limited by variability, technical failure rates, low specificity, or dependence on degradation‐prone RNA species.^2^, ^3^ A barrier to broader translation has been the lack of molecular biomarkers that are both biologically informative and analytically robust when collected non‐invasively.

TS uses an adhesive patch to collect stratum corneum material for analysis (Figure 1A). Current TS RNA assays are designed for use by dermatologists to help prioritise ambiguous pigmented lesions^2^ (Figure 1B). These assays suffer relatively high technical failure rates—up to ∼14% of tests yield invalid results^3^—likely due to degradation of the long RNA targets. As miRNAs are inherently more resistant to degradation than mRNAs^4^, we hypothesised that using more stable short RNA biomarkers could improve the reliability of TS‐based tests. We first determined that intact miRNAs are collected in TS samples and can be sensitively quantified by dPCR. TapeStation analysis confirmed that long RNA was largely degraded in TS‐derived RNA (Figure 1C). Small RNA sequencing of the preserved fraction predominantly mapped to annotated miRNAs (Figure 1D,E).

**FIGURE 1:**
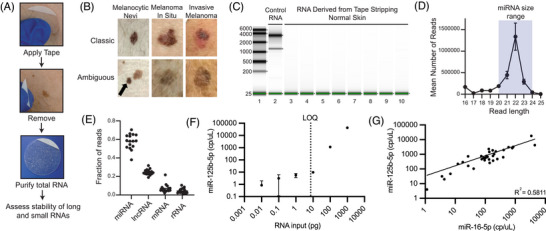
MicroRNAs are intact and detected with high sensitivity by digital polymerase chain reaction in RNA derived from Tape Stripping. (A) Example of the tape stripping (TS) collection method. (B) Visual examples of the lesions that were tape‐stripped prior to subsequent biopsy. (C) TapeStation analysis comparing high‐sensitivity RNA ladder (lane 1), high‐quality control RNA derived from a fresh/frozen patient‐derived xenograft (PDX) melanoma tumour (lane 2), and RNA derived from control TS samples (lanes 3–10). Small arrows indicate bands identified by the software as areas with measurable RNA concentrations, and green bands at the 25 base pair mark depict the internal DNA standard that serves as the technical control. (D) Average read length distributions of sequenced skin lesion TS samples from a separate cohort (GEO Series accession number GSE286566). Error bars indicate the standard error of the mean. (E) Fraction of assigned reads in Ensembl corresponding to miRNA, long non‐coding (lnc)RNA, Messenger (m)RNA, and Ribosomal (r)RNA. Bars represent mean values. (F) A dilution series of the high‐quality control PDX RNA was used to compare total RNA input into miRNA cDNA synthesis and their corresponding miR‐125b‐5p concentrations (cp/µL) detected by dPCR. Both axes were scaled to log10, and the LOQ is identified at 9.62pg of RNA input into miRNA cDNA synthesis. (G) miR‐16‐5p concentrations (cp/µL) compared to the miR‐125b‐5p concentrations (cp/µL) for a subset of the cohort. Both axes were scaled to log10, and a nonlinear fit analysis gives an R^2^ value of 0.5811.

Several miRNA expression ratios (miR‐21‐5p:miR‐125b‐5p and miR‐21‐5p:miR‐211‐5p) were previously discovered in tissue specimens and later confirmed across large meta‐analyses to consistently distinguish melanoma from nevi.^5^, ^6^, ^7^ Those studies were performed on miRNAs isolated from formalin‐fixed, paraffin‐embedded tissue sections from archival specimens. Here, we evaluate whether these previously established miRNA ratios remain diagnostic when measured non‐invasively using TS samples. We developed a multiplex dPCR assay to quantify TS‐derived miRNAs, using TaqMan Advanced miRNA probe/primer sets for three targets (miR‐125b‐5p, miR‐21‐5p, and miR‐211‐5p). An input dilution series for the miR‐125b‐5p dPCR assay revealed a limit of quantification (LOQ) of approximately 9.62 pg RNA, corresponding to ∼7.3 copies/µL (cp/µL) (Figure 1F). Outputs for miR‐125b‐5p and the commonly used loading control miR‐16‐5p were linearly related over a wide range, supporting the use of miR‐125b‐5p as a reference (Figure 1g). We excluded the miR‐211‐5p assay from quantitative analysis because its endpoint dPCR amplitude distribution lacked a discrete positive cloud, precluding objective thresholding and Poisson‐based quantification, consistent with inefficient assay performance rather than biological absence of signal (Figure 2).

**FIGURE 2:**
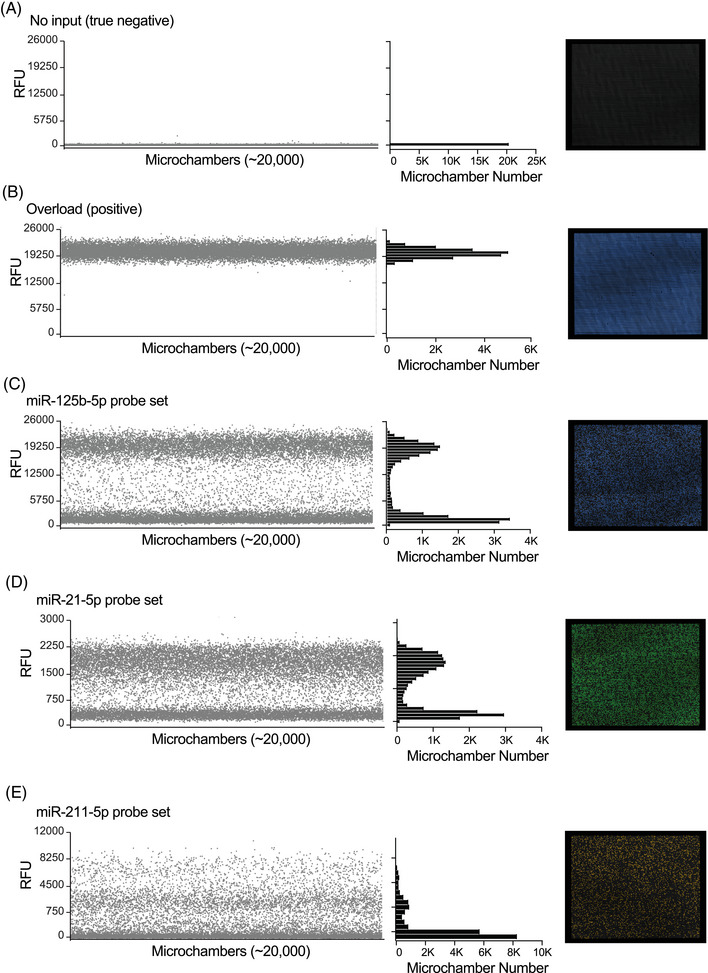
Digital polymerase chain reaction (dPCR) is unreliable with miR‐211‐5p. 1D scatterplots generated from QuantStudio Absolute Q Digital PCR Software (v6.3.5), paired histograms, and post dPCR images of the separate microfluidic arrays taken by the QuantStudio Absolute Q Digital PCR System depicting (A) A true negative No Template Control (NTC) run with the miR‐125b‐5p assay; (B) A tape stripping (TS) sample that overloaded the microfluidic array, resulting in every microchamber reporting positive for miR‐125b‐5p concentration (uniform true positive microchambers); (C) A TS sample with approximately 50/50 reported positive and negative microchambers for miR‐125b‐5p labelled with the FAM dye; (D) A TS sample with approximately 50/50 reported positive and negative microchambers for miR‐21‐5p labelled with the VIC dye; and (E) An example from miR assay optimization using normal human melanocytes that highly express miR‐211‐5p, run with miR‐211‐5p labelled with the ABY dye. Panels (C) and (D) show the expected separation of positive and negative wells. Panel (E) shows the technical failure of the miR‐211‐5p assay to result in a clear positive and negative microchamber population even when the target miRNA is well expressed.

We prospectively collected TS samples from 177 clinically suspicious pigmented lesions (in 168 patients) immediately before biopsy (Table S1). Each TS sample's miRNA profile was later matched to the lesion's histopathologic diagnosis, which included 80 MNs, 36 IMs, and 61 melanomas in situ (MIS). Patient age, sex, and anatomic distribution were consistent with expected clinical patterns across diagnostic categories (Figure 3A,B). Signals exceeded the LOQ in 96.61% of specimens (171/177) and did not correlate with cohort features (Figure S1). Consistent with prior tissue‐based studies, the miR‐21‐5p:miR‐125b‐5p ratio was significantly higher in IM than in MN (*p* < 0.0001; Figure 3C). Receiver operating characteristic analysis demonstrated discrimination between IM and MN with an area under the curve (AUC) of 79.6% (95% bootstrap confidence interval [CI] 69.3%–89.8%) (Figure 3D). Using repeated 10‐fold cross‐validation (200 iterations), a cut point yielded a sensitivity of 69.4% (95% CI 63.9%–75.0%) and specificity of 80.0% (95% CI 80.0%–82.5%) when sensitivity and specificity were equally weighted. Weighting sensitivity twice as important increased sensitivity to 75.0% with reduced specificity (66.3%). MIS, which had not been previously evaluated using this ratio, showed intermediate values between MN and IM, differing significantly from MN, while remaining lower than IM (Figure 3C). When IM and MIS were combined, discrimination from MN was preserved (Figure 3E, AUC = 70.0%, 95% CI 62.3%–77.9%), with expected trade‐offs between sensitivity and specificity depending on weighting. In the subset of samples with sufficient RNA yield to perform both assays, miRNA ratio performance compared favourably to TS mRNA markers (*PRAME* and *LINC00518*), demonstrating higher sensitivity and specificity and reinforcing the analytic robustness of the miRNA‐based approach (Figure 3F,G).

**FIGURE 3:**
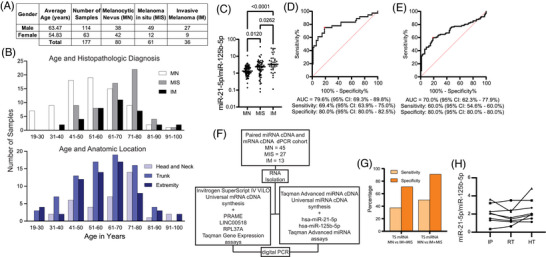
Cohort demographics and test characteristics highlight that the miR‐21‐5p:miR‐125b‐5p ratio retains the ability to distinguish melanocytic nevi from melanoma. (A) Compiled patient demographics and the types of tape stripping (TS) samples included in the analysis. (B) Patient ages compared to the number of samples that are melanocytic nevi (MN), melanomas in situ (MIS) or invasive melanomas (IM), as well as the general anatomic location from which the TS and paired biopsy originated. (C) Kruskal‐Wallis analysis comparing the miR‐21‐5p:miR‐125b‐5p calculated ratios for MN, MIS and IM to each other with *p*‐values included (MN vs. IM *p* < 0.0001; MN vs. MIS *p* < 0.0120; MIS vs. IM *p* < 0.0262). (D) Receiver operating characteristic (ROC) curve comparing the miR‐21‐5p:miR‐125b‐5p ratios for the MN TS samples (*n* = 80) to IM TS samples (*n* = 36), area under the curve (AUC) 79.6% (95% CI: 69.3%–89.8%), sensitivity 69.4% (95% CI: 63.9%–75.0%), and specificity 80.0% (95% CI: 80.0%–82.5%) when weighted equally. (E) ROC curve comparing the miR‐21‐5p:miR‐125b‐5p ratios for the MN TS samples (n = 80) to the combined MIS and IM TS samples (*n* = 97), AUC 70.0% (95% CI: 62.3%–77.9%), sensitivity 60.0% (95% CI: 54.6%–60.0%), and specificity 80.0% (95% CI: 80.0%–80.0%) when weighted equally. (F) Workflow for the subset of samples that underwent paired miRNA cDNA synthesis and mRNA cDNA synthesis and subsequent dPCR using gene and miRNA‐specific primers. (G) Calculated sensitivity and specificity for the paired miRNA cDNA and mRNA cDNA subset. (H) Calculated miR‐21‐5p:miR‐125b‐5p ratios from the normal skin cohort that underwent three separate storage conditions prior to RNA isolation. The first condition was samples that were immediately processed (IP) after collection. The second condition consisted of the samples remaining at room temperature (RT) for 3 days prior to RNA isolation. For the third condition, samples remained at room temperature for 3 days, but during that time, underwent a high temperature (HT) incubation for 12 hours at 49°C, which occurred approximately 12 hours after initial collection.

To assess assay robustness under extreme handling conditions, matched triads of tape‐strip specimens from adjacent skin were either immediately processed or processed after 3 days at room temperature with and without 12 hours at high temperature (HT, 49°C) (Figure 3H). Resulting miR‐21‐5p:miR‐125b‐5p ratios showed acceptable precision (mean CV = 11.99%), indicating stable miRNA ratio signal preservation across these conditions.

Conclusion: We demonstrate that a TS patch can collect stable miRs from skin lesions and that a miR‐21‐5p:miR‐125b‐5p ratio derived from these samples differentiates melanoma from MN with good discriminatory performance. This non‐invasive approach recapitulates a melanoma‐associated biomarker previously established in tissue within a real‐world prospective cohort, supporting its potential utility for prioritising lesions for biopsy rather than for definitive diagnosis.

Performance estimates were derived using conservative cross‐validation, and confidence intervals remain relatively wide, reflecting the sample size and underscoring the need for larger, independent cohorts to further refine these metrics. The present study focuses on melanocytic lesions, which represent the primary source of diagnostic ambiguity prompting melanoma biopsy, rather than non‐melanocytic skin tumours that are typically clinically distinct. While digital PCR provides high analytic sensitivity and precision, future validation studies will need to consider factors such as cost, throughput, reproducibility and platform selection.

Importantly, the reported sensitivity indicates that not all melanomas would be identified by this approach, emphasising that it is not intended to exclude melanoma or replace biopsy, but rather to inform prioritisation of lesions for timely clinical evaluation. With further validation, such a strategy could help improve early melanoma detection, particularly in settings with limited access to dermatologic care.

## AUTHOR CONTRIBUTIONS

*Conceptualization*: DEKKER C. DEACON, DOUGLAS GROSSMAN and ROBERT L. JUDSON‐TORRES. *Formal analysis*: CARLY A. BECKER, ROBERT L. JUDSON‐TORRES, KENNETH M. BOUCHER and CHRIS J. STUBBEN. *Funding acquisition*: DOUGLAS GROSSMAN and ROBERT L. JUDSON‐TORRES. *Investigation*: CARLY A. BECKER, DAVIDW.N. MWELWA, AMANDA Z. JIANG and ANNABELLE HUNTSMAN. *Methodology*: DOUGLAS GROSSMAN and ROBERT L. JUDSON‐TORRES. *Supervision*: ROBERT L. JUDSON‐TORRES. *Original draft preparation*: ROBERT L. JUDSON‐TORRES. *Writing—reviewing and editing*: CARLY A. BECKER, ERIC A. SMITH, DEKKER C. DEACON, DOUGLAS GROSSMAN and ROBERT L. JUDSON‐TORRES.

## CONFLICT OF INTEREST STATEMENT

The authors declare no conflict of interest.

## ETHICS STATEMENT

This study was approved by the University of Utah IRB (protocol #156786). All subjects provided written informed consent prior to participation.

## Supporting information



Supporting Information

Supporting Information

Supporting Information

## Data Availability

All primary data in the study are available upon request from the corresponding author. The sequencing data discussed in this publication have been deposited in NCBI's Gene Expression Omnibus (GEO) and are accessible through GEO Series accession number GSE286566.
