# Supplementary figures and images for: Non‐invasive adhesive patch microRNA assay recapitulates tissue biomarkers for melanoma
Source: Clin Transl Med. 2026 Feb 15;16(2):e70627. doi: 10.1002/ctm2.70627 (PMC12906660; doi:10.1002/ctm2.70627)

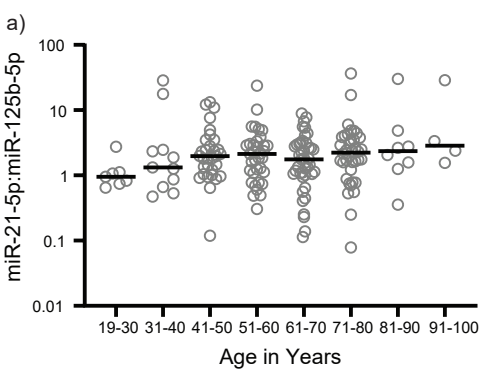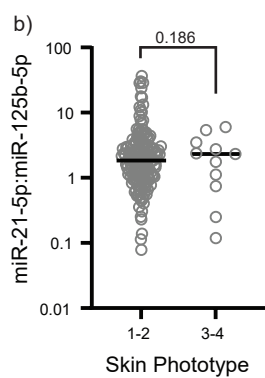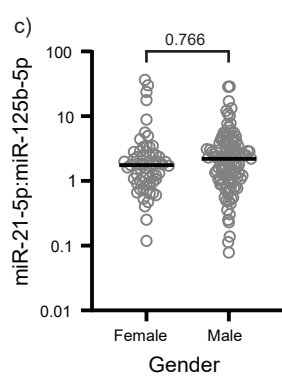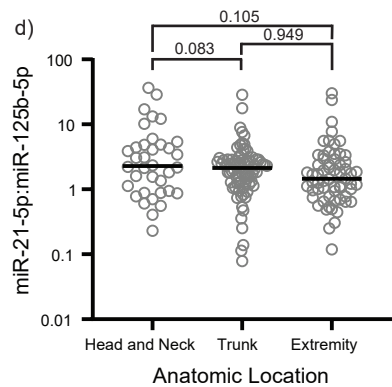

Supplement: Supplementary file 1 — Supporting Information [file CTM2-16-e70627-s002.pdf]
