# Supplementary material for: Non‐invasive adhesive patch microRNA assay recapitulates tissue biomarkers for melanoma
Source: Clin Transl Med. 2026 Feb 15;16(2):e70627. doi: 10.1002/ctm2.70627 (PMC12906660; doi:10.1002/ctm2.70627)
Supplement: Supplementary file 3 — Supporting Information [file CTM2-16-e70627-s001.docx]

**Supplementary Figure Legends**

**Supplementary Table S1. Patient demographics highlight diversity within the cohort.** Table generated in Microsoft Excel 2019 containing the gender, age, and skin phototypes for each patient along with the lesion’s histopathological diagnosis, TS and biopsy location, and sample type assigned by a physician. Also included are the miR-21-5p and miR-125b-5p concentrations (cp/uL) and the calculated miR-21-5p:miR-125b-5p ratios.

**Supplementary Figure S1. miR-21-5p:miR-125b-5p ratios binned by patient demographics (a)** Patient age bins with the corresponding miR-21-5p:miR-125b-5p ratio for each sample. Bonferroni corrected T Tests were performed using two-tailed and two-sample unequal variance parameters. No comparisons were considered statistically significant (p values<0.00179). **(b)** Skin phototypes for each sample plotted with the miR-21-5p:miR-125b-5p ratio. A T Test was performed using two-tailed and unequal variance parameters. **(c)** Patient gender for each sample plotted with the miR-21-5p:miR-125b-5p ratio. **(d)** The anatomic location of each TS sample plotted with the miR-21-5p:miR125b-5p ratio. A Bonferroni correction was applied. No comparisons were considered statistically significant (p values<0.0167).

**Supplemental Methods**

**TS sample collection and RNA isolation**

Tape-strip (TS) samples were collected from clinically suspicious pigmented lesions prior to biopsy in patients at the high-risk pigmented lesion clinic at Huntsman Cancer Institute in Salt Lake City, UT. After obtaining informed written consent, the lesion site was first cleaned with an alcohol swab and then one D-100 adhesive patch (D-Squame, Derm & Clinical) was firmly applied to the lesion, then repeatedly rotated and re-applied (such that the entire surface of the patch was used), and stored in individual vials at -80 °C. Samples corresponding to lesions that proved to be non-melanocytic (e.g., lentigo, non-melanoma skin cancer) were excluded. At this stage, lab personnel were blinded to the histopathological diagnosis for each sample until after miRNA cDNA digital PCR and mRNA cDNA digital PCR analysis. RNA was generated from all TS samples using the Qiagen miRNeasy Micro Kit as directed (Qiagen, 217084). RNA was eluted in 14uL of nuclease free water and stored at -80 °C until used for miRNA cDNA synthesis. No testing for sample quality or concentration (i.e. Tape Station) was done for the patient cohort samples prior to both miRNA cDNA and mRNA cDNA synthesis.

**miRNA cDNA synthesis and dPCR**

miRNA cDNA synthesis and amplification were completed using the Applied Biosystems™ TaqMan™ Advanced miRNA cDNA Synthesis Kit (Thermo Fisher, A28007) with 2uL of RNA input per sample. Amplified miRNA cDNA samples were then initially diluted 1:100 using a 0.1X TE buffer and stored at -30 °C.

Digital PCR was performed with the Applied Biosystems™ QuantStudio™ Absolute Q™ Digital PCR System (Thermo Fisher, A52864, Publication Number MAN0025621, Revision E.0) and the Absolute Q™ DNA Digital PCR Master Mix (5X) (Thermo Fisher, A52490). Applied Biosystems™ TaqMan™ Advanced miRNA assays (Thermo Fisher, A25576) were multiplexed to amplify two miRNAs of interest in each sample (hsa-miR-125b-5p - 477885_mir - FAM and hsa-miR-21-5p – 477975_mir - VIC). The master mix solution was made in bulk as needed, depending on the number of samples to be run that day following the protocol instructions.0.5uL of each assay were added to 2uL of master mix per reaction. Then 2.7uL of the master mix and assay solution were added to 7.3uL of diluted miRNA cDNA for each sample for a total reaction volume of 10uL. 9uL of the mixture was loaded into the appropriate well on the QuantStudio™ Absolute Q™ MAP16 Plate Kit (Thermo Fisher, A52865) and 15uL of QuantStudio™ Absolute Q™ Isolation Buffer (Thermo Fisher, A52730) was pipetted on top of each loaded sample and the wells were sealed with the provided rubber gaskets. Default dPCR conditions were provided by the Applied Biosystems™ QuantStudio™ Absolute Q™ Software (v6.3.5) with the FAM and VIC dye channels selected for analysis.

**miRNA assay optimization**

To optimize the Applied Biosystems™ TaqMan™ Advanced miRNA assays (Thermo Fisher, A25576), a series of control tests was performed to ensure quality. 10ng of high-quality RNA derived from a Normal Human Melanocyte (NHM) cell line underwent the miRNA cDNA synthesis and dilution protocol as outlined above. The NHM 1:100 dilution miRNA cDNA was tested with each miRNA cDNA assay (hsa-miR-125b-5p - 477885_mir – FAM, hsa-miR-21-5p – 477975_mir – VIC, and hsa-miR-211-5p - 478507_mir – ABY) in triplicate to see if the assay produced a clear positive population and clear negative population. Then the same samples were tested with various combinations of the three assays in triplicate to determine if they were capable of reproducing the same results when run as a multiplex instead of individually. We saw that hsa-miR-211-5p was incapable of producing a clear positive and negative population of microchambers both individually and when grouped with the other assays, and so it was removed from our study. hsa-miR-21-5p and hsa-miR-125b-5p were able to consistently reproduce their reported concentrations when run individually and multiplexed.

The LOQ analysis for the hsa-miR-125b-5p assay used a dilution series of high-quality RNA derived from a fresh/frozen Patient Derived Xenograft (PDX) melanoma tumor tissue. The dilutions ranged from 1ng to 0.01pg of input into the miRNA cDNA synthesis protocol described above. These samples were then diluted as described above and dPCR was run in quadruplicate as described above. To ensure that there was no contamination of the assays or nuclease free water used for all samples tested with the miR assays, a No Template Control (NTC) well, where the 7.3uL of cDNA sample was replaced with nuclease free water, was included once every two-three dPCR plates.

**Digital PCR quality control and inclusion in analysis pipeline**

After dPCR, an image was captured of the microfluidic array for each dye channel selected (FAM, VIC, ABY, and/or CY5) as well as the quality control ROX dye included in the master mix. These images were then compared to images of the microfluidic array taken at various timepoints within the run, and the differences in observed fluorescence were then used to generate the 1D rainfall plots for each sample and dye used. The final array images were checked for each sample and dye by the technicians to ensure even sample loading and to compare to the internal quality control ROX dye. In order to be included in the analysis, each sample must have passed the software’s own quality control analysis, not overloaded any assay, and exhibited array images that indicated even loading and minimal microchamber omission.

**miRNA cDNA dPCR analysis**

For miRNA dPCR analysis, the Auto Sample fluorescence threshold was set by the software for each sample on the 1D rainfall plot and manually adjusted if necessary. After the fluorescence threshold was set, the software generated a cp/uL concentration for each sample for both hsa-miR-125b-5p and hsa-miR-21-5p. We then divided the hsa-miR-21-5p concentration by the hsa-miR-125b-5p concentration to obtain the ratio used in analysis. Some samples upon the first round of dPCR were too concentrated for one or both assays and flagged as overloaded by the software. These samples were subsequently diluted further in 0.1X TE and reran until a positive microchamber count of less than 16,000 was achieved for the overloaded assay per Thermo Fisher guidelines.

**Paired subset mRNA cDNA synthesis and dPCR**

mRNA cDNA for the samples in the paired miRNA cDNA and mRNA cDNA digital PCR subset were generated from the same TS RNA. mRNA cDNA synthesis was conducted using Invitrogen™ SuperScript™ IV VILO™ without ezDNase kit and protocol (Thermo Fisher, 11756050). RNA input volumes varied for each sample and ranged from 2uL to 10uL depending on RNA amount available. mRNA cDNA was stored at -30 °C. dPCR was performed with the Applied Biosystems™ QuantStudio™ Absolute Q™ Digital PCR System (Thermo Fisher, A52864, Publication Number MAN0025621, Revision E.0) and the Absolute Q™ DNA Digital PCR Master Mix (5X) (Thermo Fisher, A52490). Three Taqman Gene Expression Assays (Thermo Fisher, 4331182) were multiplexed for each reaction to amplify genes of interest in the samples *RPL37A*, *LINC00518*, and *PRAME* (assay information below). Per protocol instructions, 0.5uL of each assay were added to 2uL of master mix and 4.2uL of water for each reaction. Then 7uL of the master mix, assays, and water mixture were added to 3uL of mRNA cDNA sample for a total volume of 10uL. 9uL of this reaction mixture was loaded into the appropriate well on the QuantStudio™ Absolute Q™ MAP16 Plate Kit (Thermo Fisher, A52865) and 15uL of QuantStudio™ Absolute Q™ Isolation Buffer (Thermo Fisher, A52730) was pipetted on top of each loaded sample. The wells were sealed with the provided rubber gaskets and default dPCR conditions were provided by the software with the VIC, ABY, and CY5 channels selected for analysis. After dPCR, concentrations (cp/uL) for amplified genes were generated for *RPL37A* (Hs01102345_m1 - CY5), *LINC00518* (Hs00332749_m1 – VIC), and *PRAME* (Hs01022301_m1 – ABY) using the Applied Biosystems™ QuantStudio™ Absolute Q™ Software (v6.3.5).

**mRNA assay optimization**

In order to ensure the Taqman Gene Expression Assays (Thermo Fisher, 4331182) were reliable, a series of control tests were performed to ensure quality and establish dPCR thresholds. 10ng of high-quality RNA derived from fresh/frozen breast reduction tissue and a fresh/frozen PDX melanoma tumor tissue underwent the mRNA cDNA synthesis as outlined above. For the purposes of this experiment, the breast reduction cDNA served as our negative control, and the PDX melanoma tumor cDNA was our positive control. Both samples were prepared as stated above and tested with each assay individually in quadruplicate. This allowed us to confirm: 1) that the normalizing assay (*RPL37A*) could be reproducibly detected in both sample types and 2) that the melanoma associated genes (*LINC00518* and *PRAME*) were only detected in the PDX tumor and not the breast reduction tissue.

Once this was confirmed, the PDX tumor sample and breast reduction samples were used to optimize the multiplexing of the three assays and run in quadruplicate. Relative Fluorescence Unit (RFU) thresholds for the three gene expression assays were set based on these control samples and placed under where the positive microchamber populations were consistently present. The *RPL37A* gene expression assay had a fluorescence threshold set at 1,000 RFU, the *LINC00518* gene expression assay was set at 1,000 RFU, and the *PRAME* gene expression assay was set to 4,000 RFU. No microchambers were positive for our breast reduction cDNA when run with the melanoma associated genes, *LINC00518* and *PRAME*. This allowed us to generate a set threshold to compare all TS samples to for if the gene was expressed or not expressed in each sample.

An NTC well (as described in miR dPCR optimization) was also included once every two-three dPCR runs to ensure the assays and nuclease free water had not become contaminated.

**mRNA cDNA dPCR analysis**

After dPCR and the sample passed the quality control process as described above, every sample had RFU thresholds adjusted to match those set by the controls for each assay. After the thresholds were adjusted, the software calculated concentrations (cp/uL) for each assay within each sample which were used to make binary calls for gene expression.

**Test characterization & statistical analyses**

For the miRNA assay, hsa-miR-125b-5p was used as the input control, such that samples that had expression less than 7.3 cp/uL were considered a failed assay (6/177 samples failed). A miR-21-5p:miR-125b-5p ratio above 2.87 was called positive for melanoma (MIS or IM). This miR ratio was chosen as the ratio that minimized the number of False Negatives for the MN category. For the RNA assay, *RPL37A* was used as the input control, such that no expression would be considered a failed assay (4/85 samples failed). For successful RNA assays, the detection of either *LINC00518* or *PRAME* was considered positive for melanoma (MIS or IM). For the purposes of recommending biopsy, failed runs in either category were also considered ‘positive’, as a failed test of a suspicious lesion would necessitate default to the standard of care (biopsy). Sensitivity and Specificity for the ROC curves were calculated in R (v4.5.1) using package pROC. Ten-fold cross-validation was used to estimate the sensitivity and specificity at two cut points: the optimal cut point giving equal importance to sensitivity and specificity, and the optimal cut point giving twice the importance to sensitivity as to specificity. Cross-validation was repeated 200 times to produce a median estimate and a confidence interval. All other statistical analyses were generated using Prism version 10 software (Graphpad) or Microsoft Excel 2019. For the paired miRNA cDNA and mRNA cDNA subset, sensitivity was calculated as True Positives ÷ (True Positives + False Negatives) and specificity as True Negatives ÷ (True Negatives + False Positives). P values <0.05 were considered statistically significant unless otherwise stated.

**Normal Skin TS Sample Collection, RNA Isolation, and TapeStation Analysis**

Control TS samples were collected using the same method as described above, but they did not undergo a biopsy. For each participant, three adjacent TS samples were taken from lesion free skin on the upper inner arm and three TS samples were taken from lesion free skin on the upper back. One sample from the upper arm and one from the upper back were subjected to one of three conditions prior to RNA isolation. The first condition was immediate RNA isolation post TS collection (IP). The second condition consisted of the samples sitting in tubes at room temperature for 72 hours and then undergoing RNA isolation (RT). The third condition was that the samples sat in tubes for 72 hours at room temperature, but approximately 12 hours after TS collection, the tubes were placed in an incubator at 49°C for 12 hours and then returned to room temperature (HT) until RNA isolation at 72 hours post TS collection. RNA was isolated using the method described above and stored at -80 °C. The control RNA from lane 2 in the Tape Station analysis was derived from a separate fresh/frozen PDX melanoma tumor using the Qiagen AllPrep DNA/RNA Mini Kit (Qiagen, 80204) protocol as written. All RNA samples were sent to the High-Throughtput Genomics core at Huntsman Cancer Institute for quality and concentration testing using the High Sensitivity RNA ScreenTape Analysis (Agilent, 5067-(5579-5581)) on the Agilent 2200 TapeStation System. Small arrows to the right of each sample lane indicate measured RNA within the sample and a concentration is reported. Absence of bands indicates no detected RNA, and green bands at 25 base pairs depict the internal DNA standard that serves as the technical control.

**miR sequencing**

Small RNA sequencing was performed on an Illumina NovaSeq 6000 instrument as described previously^8^ in a separate Basal Cell Carcinoma TS cohort which underwent the same collection and RNA extraction process as outlined above. Briefly, libraries were prepared from approximately 10 uL of total RNA eluate (concentration too low to be quantitated) and library size was selected to enrich for adapter-ligated molecules encoding small RNA species.

**Analysis of miR sequencing**

Alignment of sequencing data was performed as previously described in Fastner et al^8^ on the separate Basal Cell Carcinoma cohort. Briefly, the trimmed reads were aligned to a reference human database optimized for shorter miR reads and the mapped reads were assigned to annotated genes in Ensembl and to miRs in miRbase release 22.1.^9^ Differentially expressed miRs were identified using a 5% false discovery rate, and the regularized log values from the top 500 most variable miRs were used for principal components analysis.

**Patient Demographics analysis**

We compared the calculated miR ratios to the associated patient ages, skin types, gender, and locations within the Patient Demographics data provided in Supplementary Figure S1. For each comparison, we did single T tests in Microsoft Excel 2019 comparing each category to each other choosing two-tailed and two-sample unequal variance parameters. For the graphs where multiple comparisons were needed, an adjusted p value was calculated using Bonferroni correction. For graphs that did not need Bonferroni correction, p values<0.05 were considered statistically significant.
